# Negative symptoms - a real unmet need

**DOI:** 10.1192/j.eurpsy.2022.2044

**Published:** 2022-09-01

**Authors:** I. Soares Da Costa, R. Moreira

**Affiliations:** CHUSJ, Psychiatry, Porto, Portugal

**Keywords:** negative symptoms, schizophrénia

## Abstract

**Introduction:**

Schizophrenia is frequently a chronic and disabling disorder, characterized by heterogeneous positive and negative symptoms. Negative symptoms are a major health concern and a core component of schizophrenia that account for a large part of the long-term disability and poor functional outcomes in patients with the disorder. Adequate treatment would mean important progress and Distinguishing primary from secondary negative symptoms may inform about treatment options.

**Objectives:**

Objective: to provide information that may be useful for clinicians treating patients with negative symptoms of schizophrenia.

**Methods:**

We searched Pubmed and Cochrane Library database for english language articles.

**Results:**

Negative symptoms are a core component of schizophrenia that account for a large part of the long-term disability and poor functional outcomes. Negative symptoms are common in schizophrenia; up to 60% of patients may have prominent clinically relevant negative symptoms that require treatment. Negative symptoms can occur at any point in the course of illness, although they are reported as the most common first symptom of schizophrenia. Negative symptoms can be primary symptoms, which are intrinsic to the underlying pathophysiology of schizophrenia, or secondary symptoms that are related to psychiatric or medical comorbidities, adverse effects of treatment, or environmental factors. Negative symptoms clearly constitute an unmet medical need in schizophrenia, and new and effective treatments are urgently needed.

**Conclusions:**

Clinically relevant negative symptoms of schizophrenia need to be recognized, assessed, and as well managed as possible in order to achieve improved outcomes for patients. More studies are needed to establish the better approach to negative symptoms.

**Disclosure:**

No significant relationships.

